# Transfer-Learning Deep Raman Models Using Semiempirical Quantum Chemistry

**DOI:** 10.1021/acs.jcim.5c00513

**Published:** 2025-06-18

**Authors:** Jawad Kamran, Julian Hniopek, Thomas Bocklitz

**Affiliations:** † Institute of Physical Chemistry, 9378 Friedrich Schiller University Jena, Helmholtzweg 4, 07743 Jena, Germany; ‡ Department of Photonic Data Science, 40096 Leibniz Institute of Photonic Technology, Albert-Einstein-Straße 9, 07745 Jena, Germany

## Abstract

Biophotonic technologies such as Raman spectroscopy are powerful tools for obtaining highly specific molecular information. Due to its minimal sample preparation requirements, Raman spectroscopy is widely used across diverse scientific disciplines, often in combination with chemometrics, machine learning (ML), and deep learning (DL). However, Raman spectroscopy lacks large databases of independent Raman spectra for model training, leading to overfitting, overestimation, and limited model generalizability. We address this problem by generating simulated vibrational spectra using semiempirical quantum chemistry methods, enabling the efficient pretraining of deep learning models on large synthetic data sets. These pretrained models are then fine-tuned on a smaller experimental Raman data set of bacterial spectra. Transfer learning significantly reduces the computational cost while maintaining performance comparable to models trained from scratch in this real biophotonic application. The results validate the utility of synthetic data for pretraining deep Raman models and offer a scalable framework for spectral analysis in resource-limited settings.

## Graphical abstract

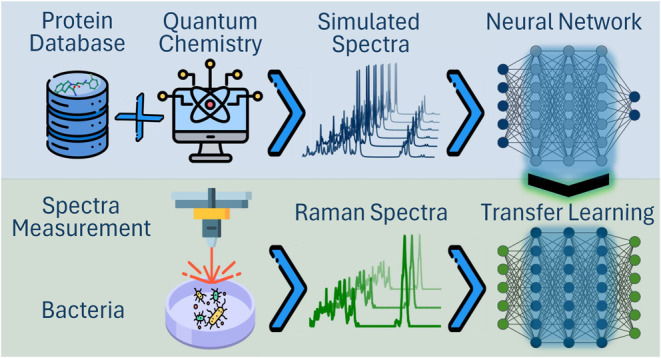

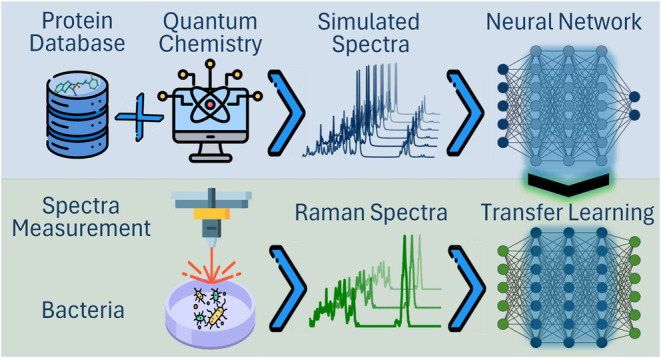

## Introduction

Raman spectroscopy is a powerful analytical technique known for its remarkable ability to provide highly specific molecular information. It has found applications in a wide range of scientific disciplines due to its intrinsic molecular specificity, nondestructive character, minimal sample preparation requirements, and the capability to analyze samples in their native environments. Its utility spans diverse fields such as material identification, chemical analysis, and biomedical research.^1^ Particularly exciting are biomedical applications, where Raman spectroscopy shows great promise for drug discovery and development,^2^ chemical bioanalysis,^3,4^ tissue classification for gastrointestinal diseases^5^ and skin lesions^6^ and numerous other medical research areas. Bacterial infections continue to pose a significant threat to human health, causing a wide range of illnesses that can lead to severe complications and mortality. The rise of antimicrobial resistance (AMR) has further exacerbated this issue, rendering many standard treatments ineffective and emphasizing the urgent need for rapid and accurate diagnostic tools.^7,8^ The review on antimicrobial resistance suggests that AMR might cause an alarming 10 million deaths annually by 2050.^9^ In this context, Raman spectroscopy has the potential to be a powerful technique for bacterial classification and identification, offering noninvasive, label-free, and rapid analysis capabilities for point-of-care applications.^10–12^ However, distinguishing between Raman spectra of chemically or structurally similar compounds, or closely related biological entities, presents a significant challenge. This difficulty is further compounded by the high-dimensional nature of Raman spectral data, along with issues related to standardizing instrumentation and calibration procedures.^13^ These challenges often limit the effectiveness of conventional data analysis strategies. In this context, we distinguish between traditional chemometric methods—such as principal component analysis (PCA), linear discriminant analysis (LDA), partial least squares (PLS), support vector machines (SVM), random forests, and k-nearest neighbors (k-NN), which depend on manually extracted features from preprocessed spectra. Collectively, these approaches can be categorized within classical machine learning which is characterized by a seperation between feature engineering and model inference. In contrast, deep learning techniques integrate these two steps within a single framework. While traditional chemometric methods have been widely used in spectroscopy, their ability to generalize is limited when dealing with complex, noisy, or nonlinear spectral data. In contrast, deep learning models can learn hierarchical representations directly from raw input, making them particularly well-suited for capturing subtle patterns and interactions in spectral data. As a result, deep learning is increasingly favored for achieving robust and scalable classification performance in spectroscopic applications.

Deep learning models have demonstrated their remarkable efficacy in extracting valuable insights from complex data, including spectral data^14^ where they have been used for tasks such as component identification in mixtures,^15^ detection and identification of microbial contamination,^16^ spectrum reconstruction^17^ and rapid detection of pathogenic bacteria.^18^ Deep learning enables the development of computational models composed of multiple processing layers, allowing for the learning of data representations at various levels of abstraction.^19^ This is particularly advantageous as classical machine learning models may struggle to capture the intricacies of nonlinear processes inherent in such data. These artificial neural networks (ANNs) have had a profound impact across multiple scientific disciplines. However, these models typically demand substantial data sets for training, validation, and testing.^20,21^ In the context of Raman Spectroscopy this leads to inherent challenges arising from the low sensitivity of the Raman effect and the time-consuming, often costly nature of gathering extensive spectra in the medical domain. These limitations typically severely limit the size of training data sets, which commonly leads to issues like overfitting, over estimation and limited model generalizability.^22,23^

We attempt to tackle the issues posed by the scarcity of extensive Raman spectral data sets by exploring the application of transfer-learning methods. Transfer learning is a powerful machine learning approach that leverages pretrained models to address new tasks, significantly reducing computational costs and training time compared to building a model from scratch. This approach is particularly valuable when working with limited data,^24,25^ as training deep neural networks from scratch on small data sets often leads to overfitting. Rather than starting from scratch, models first learn general patterns and representations from a large, diverse data set. This prelearned knowledge is then fine-tuned for specific tasks, reducing the demand for extensive task-specific data and accelerating the learning process. Similar to convolutional networks in computer vision, where early layers detect low-level features such as edges and deeper layers capture more abstract patterns,^26,27^ the feature extractor part of the CNN in spectral models learns progressively complex representations by stacking multiple convolutional layers. In the context of vibrational spectroscopy, initial layers may respond to broad spectral bands or peak positions, while deeper layers integrate these into higher-level features relevant to molecular or class-specific structure.^28,29^ This hierarchical representation allows the network to abstract chemically meaningful patterns from raw spectra, making deep learning particularly suited for data-driven spectral classification tasks. Transfer learning capitalizes on the similarity of these lower and intermediate-level features across domains. Consequently, instead of retraining the entire model, transfer learning focuses on fine-tuning the top layers of a pretrained network to adapt it for the specific task at hand. While transfer learning reduces the need for task-specific data, it still requires large pretraining data sets, which are impractical to obtain experimentally. One approach is to mathematically generate spectra using Lorentzian shapes; however, this method has limitations, including the difficulty of simulating realistic spectra and the lack of direct relation to real-world tasks.

To address the limitations posed by the scarcity of large, labeled Raman spectral data sets—particularly in biomedical contexts—we propose a transfer-learning framework that leverages synthetic spectral data generated in silico. Specifically, we use semiempirical quantum chemical methods to generate a large and diverse library of simulated vibrational spectra corresponding to molecules with varied functional groups. These synthetic spectra serve as a source domain for pretraining a deep learning model, allowing it to learn generalized spectral representations in a data-rich environment.

The pretrained model, based on a one-dimensional (1D) convolutional neural network (CNN), is then fine-tuned on a smaller, experimentally acquired Raman data set consisting of bacterial spectra. This fine-tuning step enables the model to adapt its learned features to the specific characteristics of the target domain, such as biological variability, noise, and instrumentation effects. By separating the representation learning phase from the task-specific adaptation, the proposed approach reduces overfitting, improves generalizability, and significantly lowers the computational cost compared to training a model from scratch on limited data.

This study presents a complete pipeline—from synthetic data generation to model pretraining and model transfer—aimed at demonstrating the feasibility and utility of using artificial Raman spectra for transfer learning. Our results are evaluated against both traditionally trained deep learning models and baseline methods such as PCA-LDA to assess the comparative benefits of the transfer-learning strategy. In doing so, we aim to establish a scalable, reproducible foundation for applying data-efficient deep learning in Raman-based biosensing applications.

## Methods and Experimental Setup

The workflow illustrated in Figure 1 outlines the process of synthetic spectra generation and transfer learning for bacterial classification tasks. Initially, small protein molecules are used to generate calculated line spectra using the GFN2-xTB2 method. These line spectra are broadened with a Voigt profile to simulate realistic spectral lines, followed by the introduction of artifacts such as Gaussian noise and background signals. The result is a set of simulated spectra, which serve as the pretraining data for deep learning models. These pretrained models are then fine-tuned using a bacterial data set to improve classification performance. This workflow enables efficient transfer learning by leveraging systematically generated synthetic data, providing flexibility in adjusting spectra properties to suit different tasks.

**Figure 1:**
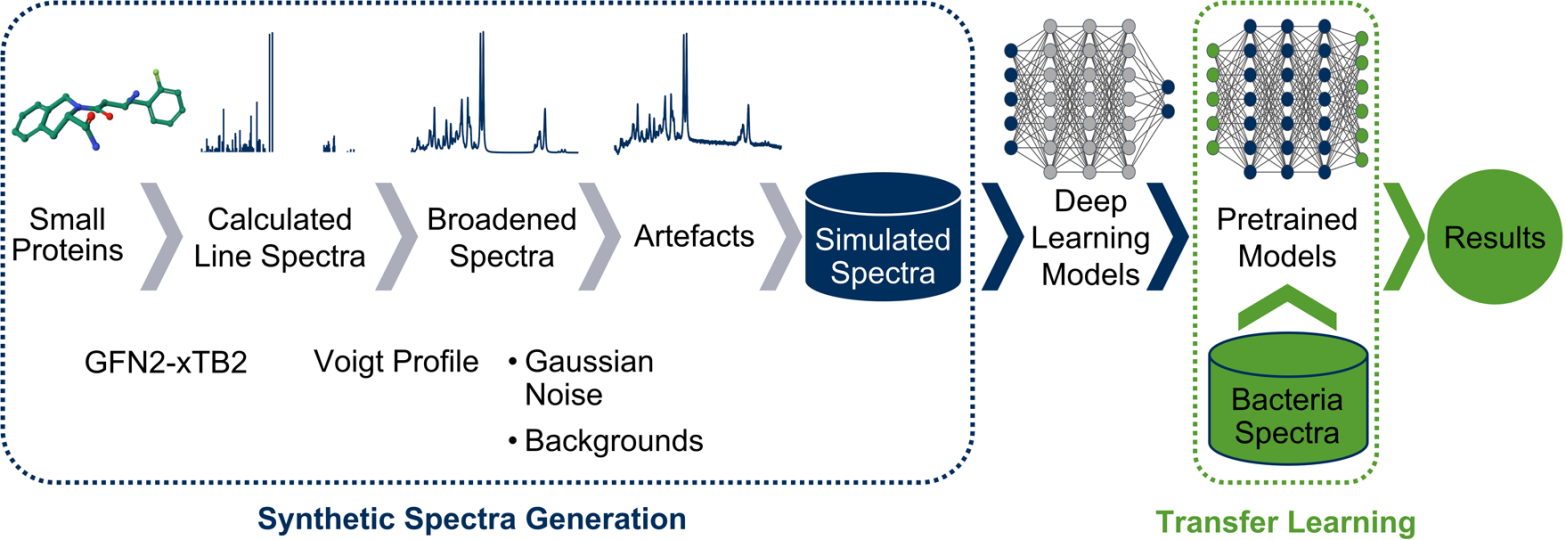
Overview of the synthetic data set generation workflow for transfer learning. Small protein structures are used to calculate line spectra with GFN2-xTB2, which are broadened using Voigt profiles. Artifacts such as Gaussian noise and background signals are introduced to create simulated spectra. These spectra are then used to pretrain deep learning models, which are subsequently fine-tuned on bacterial data sets for transfer learning, resulting in the final model performance evaluation and comparison to other approaches.

### Artificial Spectra Generation

For model pretraining, a synthetic data set of vibrational spectra was generated using semiempirical quantum chemistry methods. Approximately 19,000 species were used for a small molecule set from the Worldwide Protein Database (wwPDB).^30^ Molecules were selected based on the availability of three-dimensional (3D) geometries and successful spectral calculation. Species lacking valid geometry files or those for which the quantum chemical calculation did not converge were excluded. Additionally, only functional groups with at least 50 occurrences were retained, resulting in 51 label categories and further reducing the number of eligible molecules. The vibrational line spectra were then calculated using the Geometries, Frequencies, and Non-Covalent Interactions with the eXtended Tight-Binding (GFN2-xTB)^31^ method. Ab initio quantum mechanical methods like Hartee-Fock (HF), configurational interaction (CI), and density functional theory (DFT) provide highly accurate results but are computationally expensive for systems larger than a few hundred atoms. Classical force field (FF) methods offer faster calculations but introduce larger errors.^32^

GFN2-xTB offers a middle ground by using the tight-binding approximation with additional terms for electrostatic and dispersion effects, resulting in reasonable accuracy with significantly reduced computational cost.^33^ This makes GFN2-xTB particularly useful for large systems such as proteins and DNA. For smaller molecules, higher accuracy could be achieved with more computationally intensive methods but given the need to perform calculations on around 19,000 molecules, GFN2-xTB was selected for its efficiency. Although not as accurate as DFT, it provides a practical and reliable tool for investigating vibrational spectra, especially in large-scale studies where computational resources are a key consideration.^34^

GFN2-xTB was performed using the *xtb* package, Version 6.5.1, developed by the Grimme Group at the University of Bonn.^35,36^ For each molecule a simple geometry optimization followed by frequency calculation was performed using the real-world geometries obtained from the PDB as the starting geometry and the *--ohess* option in *xtb. The --vtight* optimization convergence criterium $\left(\frac{E_{conv}}{E_{h}}=5\cdot 10^{-6},\frac{G_{conv}}{E_{h}\cdot \alpha }=2\cdot 10^{-4}\right)$ was used to ensure a sufficiently converged geometry for frequency calculation. The resulting calculated vibrational frequencies were again checked for the presence of imaginary modes by parsing the resulting output files. If an imaginary mode was present, the molecule was rejected from the training data.

An example of a calculated line spectrum from one of the molecules in the data set is given in Figure 2A. These line spectra were subsequently broadened to mimic experimental spectra.

**Figure 2:**
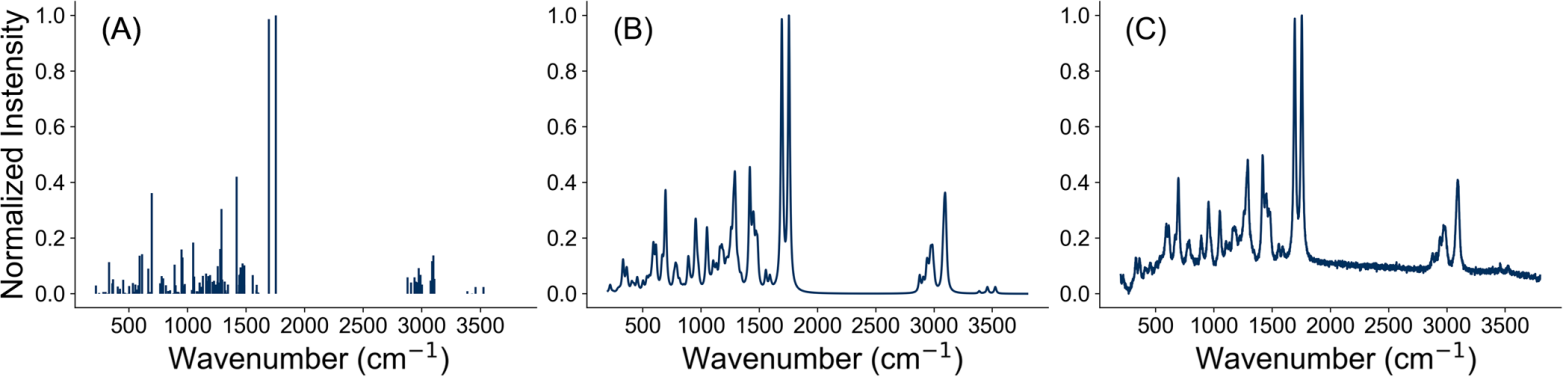
Various stages in the generation of artificial spectra. (A) A calculated line spectrum, representing spectra peaks from theoretical calculations of the GFN2-xTB model. (B) A broadened line spectrum, where peaks are convolved with a Voigt profile to simulate realistic spectral shapes. (C) A spectrum with added artifacts, including Gaussian noise and background signals, to mimic experimental conditions.

In spectroscopy, the line shape is determined by the intrinsic properties of the underlying phenomenon and is further broadened due to thermal, Doppler, and collisional effects.

The line profile of Raman spectra is, according to the molecular vibrational and rotational theories, intrinsically Lorentzian.^37^ However, the line profile measured by the spectrometers is also influenced by sources of broadening which follow Gaussian distribution e.g., Doppler effects. Therefore, the overall broadening can be modeled using a Voigt profile.^38^ The Voigt profile, denoted as *V* (*x*; σ, γ), is defined as the convolution of a Gaussian function *G* (*x*; σ) and a Lorentzian function *L* (*x*; γ). The profile was mathematically calculated using Kielkopf approximation^39^ and varying the Gaussian-to-Lorentzian ratio. A complete mathematical derivation can be found in the Supporting Information (A).

The parameters for the Voigt profile were selected from a range encompassing various Lorentzian-to-Gaussian percentages (*k*), including values of 0, 10, 30, 50, 70, and 99% and the full width at half maximum (fwhm) with 12, 16, 20, 24, 28, and 32 cm^–1^. A fixed width was applied across the entire spectrum, based on the assumption that broadening effects introduced by the experimental setup dominate the observed peak widths. These experimental effects, such as laser line width, detector resolution, and optical limitations, are consistent for all bands under the same measurement conditions. While minor fluctuations in intrinsic molecular broadening may exist, they are typically not resolvable within the spectral resolution of the system.^40^ The fwhm outside this range was deemed too narrow/wide to be observed in most experimental scenarios. The Voigt profile is applied to each data point in a line spectrum, within the wavenumber range of 200 to 3800 cm^*–*1^, transforming them into broader spectral peaks, modeling the inherent broadening effects that can occur in various spectroscopic measurements. These broadened profiles are summed together, resulting in the final spectrum which is subjected to *L*_2_ normalization.

This resulted in generation of 42 total broadened spectra for each line spectra. An example of such broadened spectrum from the line spectrum is given in Figure 2B.

### Artifacts

To create a synthetic data set that closely resembles real spectra, noise and background artifacts were deliberately introduced to the broadened line spectra.

A database of spectral backgrounds extracted from around 8000 experimentally measured Raman spectra was used as a starting point. The experimental spectra chosen were recorded on various Raman spectrometers in unrelated studies. They consist of measurements of polymers, proteins, photocatalytic systems, tissue samples and bacteria. The spectrometers range from highly confocal Raman microscopes with excitation wavelength of 514 nm to a Fourier-Transform Raman spectrometer with an excitation wavelength of 1064 nm. The data set therefore provides a large variety of potential backgrounds and artifacts encountered in Raman spectroscopy and should be representative for the shapes of backgrounds encountered in real-world experimental spectra.

The experimental backgrounds were extracted using the SNIP algorithm,^41^ a simple background estimation algorithm that is commonly used in Raman spectroscopy. 50 iterations were performed for each spectrum, which is considered a good baseline value for estimating backgrounds in Raman spectroscopy. A unique background was assigned to each spectrum in the data set. This was achieved by randomly selecting a background from the database for each broadened spectrum and adding it with a strength chosen from within a realistic range. The strength was determined by a division factor, randomly selected between 40 and 90% of the selected normalized background, to approximate the visual appearance of the backgrounds in real spectra. The normalized average background and the standard deviation is given in Figure 3.

**Figure 3:**
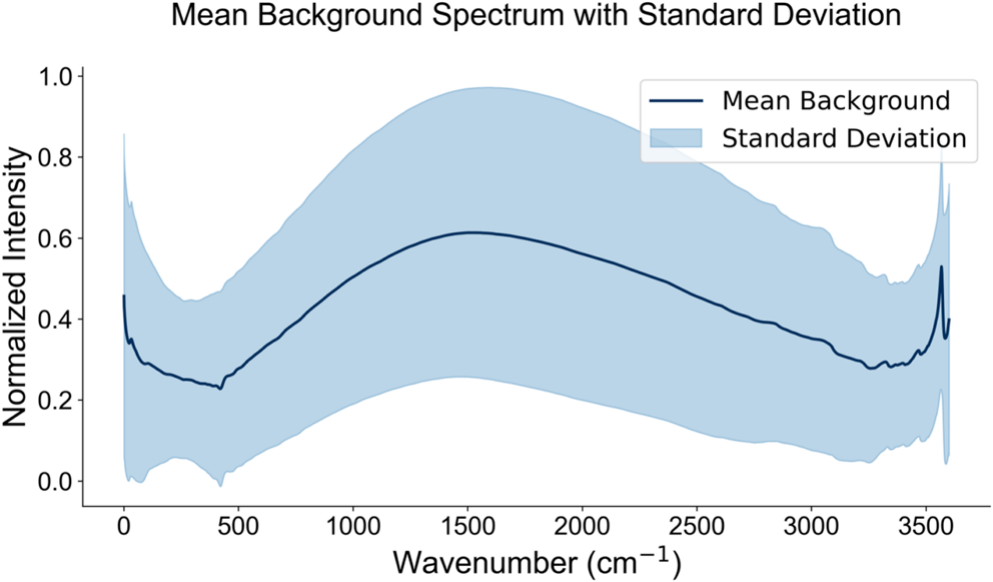
Average background spectrum with standard deviation. The solid line represents the mean normalized intensity across 8000 background spectra, while the shaded region indicates the standard deviation. Backgrounds were taken from unrelated experimental data and added to the simulated spectra with varying strengths.

Due to inherent weakness of the Raman effect and the intrinsic properties of the charge coupled device (CCD) sensors, an experimental Raman spectrum suffers from various sources of noise such as detector read noise, shot noise and dark current noise.^42^ Most of these noise sources follow a Poisson distribution, however, it is possible to approximate them with a Gaussian distribution if the spectral irradiance i.e., the mean photoelectron counts registered in the CCD are high enough.^43^ Therefore, for the simulated spectra, the noise was modeled with a single additive Gaussian distribution and randomly added to each spectrum. Specifically, a Gaussian noise vector was generated using a random number between 10^–4^ and 10^–3^. The resulting noise was subsequently added to the combined spectrum, introducing subtle fluctuations to mimic noise typically encountered in experimental data. A broadened spectrum with noise and background artifacts is shown in Figure 2C.

After the addition of background and noise the spectra were renormalized to the 0–1 scale.

### Functional Group Labels

For the synthetic spectra, functional group labels were generated using pyCheckmol^44^ software package from the SMILES strings included in the PDB data set. Each spectral sample within the data set is associated with a subset of functional group labels, selected from a total of 51 different labels available for assignment. On average, each sample is annotated with approximately four distinct labels. The distribution of these labels exhibits variability, with differing frequencies attributed to individual labels as seen in Figure 4. A complete chart of all functional group labels present in the database and their distribution can be found in the Supporting Information (B): Figure S1.

**Figure 4:**
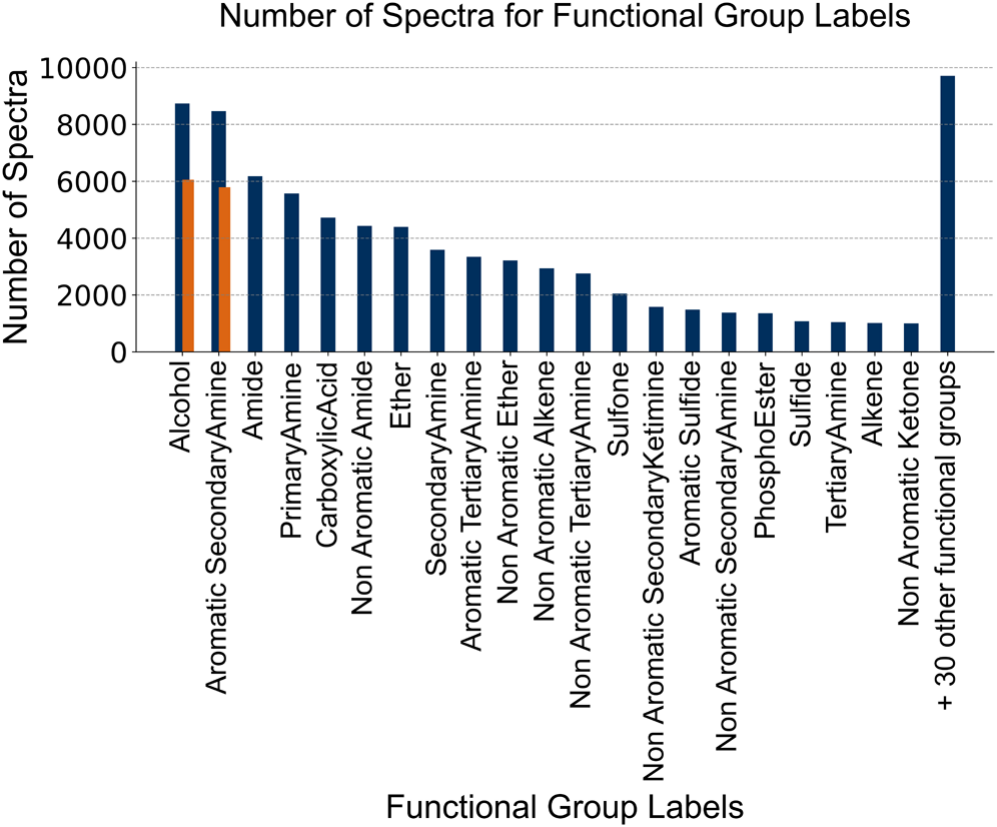
Distribution of functional group labels in the synthetic data set, showing the number of spectra per group. The two target classes used for pretraining, alcohol and aromatic secondary amine, are highlighted in orange, while the remaining functional groups provide additional context for the classification task. The “+30 other functional groups” category consolidates spectra from less frequent (<1000 spectra) classes.

The deep learning model was pretrained for a binary classification task, where the model provides the probability of belonging to one of two specific functional group classes. “Alcohol” and “Aromatic secondary amine” were chosen for this task, as they form the largest mutually exclusive pair within the data set and are relatively balanced, occurring in 11,847 unique molecules (6057 labeled as “alcohol” and 5790 as “aromatic secondary amine”). As 42 spectra for each species were generated using multiple parameters (as outlined in the Artificial Spectra Generation section) the final data set consisted of 497,616 spectra. This binary setup was selected to keep the pretraining task simple and controlled, allowing an initial evaluation of learning from artificial spectra before moving to more complex settings. Attempts at multilabel classification using all 51 functional groups proved unreliable due to severe class imbalance and overlapping spectral features, making interpretation and convergence difficult.

For binary classification, all other labels in the selected samples were ignored, and only the presence of these two functional groups was used to assign class membership. However, it is important to note that the spectra themselves still contain vibrational contributions from other functional groups present in each molecule—ranging from none to several—out of the 51 available categories. The model therefore learns to associate relevant patterns with the target labels while ignoring unrelated spectral contributions.

The mean spectrum of the spectra containing each functional group label is shown in Figure 5.

**Figure 5:**
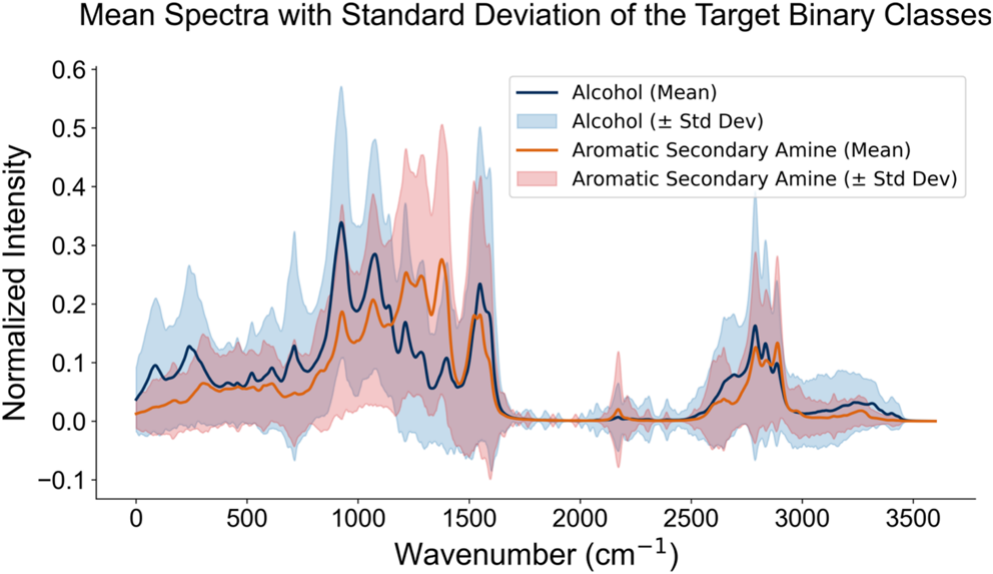
Mean spectra with standard deviation for the target binary classes, alcohol (blue) and aromatic secondary amine (red). The spectra represent the mean normalized intensity but contain contributions from additional functional groups present in the data (see Figure 4), which influence the observed variability.

### Experimental Bacteria Data Set

In order to test performance of the pretrained models, an experimentally acquired data set consisting of Raman spectra from various bacterial species was used.

This data set consists of a total of 5420 spectra measured across 9 independent batches of six different bacterial species: DSM 423, DSM 2687, DSM 5190, DSM 20649, DSM 20316, and DSM 20261. These bacterial species were sourced from the Deutsche Sammlung von Mikroorganismen and Zellkulturen GmbH (DSMZ). Details regarding these bacteria cultures, spectral data set preparation, including preprocessing steps such as despiking, wavenumber calibration for peak position correction, background correction, smoothing, and normalization, can be found in the article Ali et al.^45^ The mean spectra of different strains of bacteria in the data set is given in Figure 6 providing a visual summary of interclass spectral similarities and overlaps. This overview helps illustrate the challenge of distinguishing between closely related classes based on Raman signatures.

**Figure 6:**
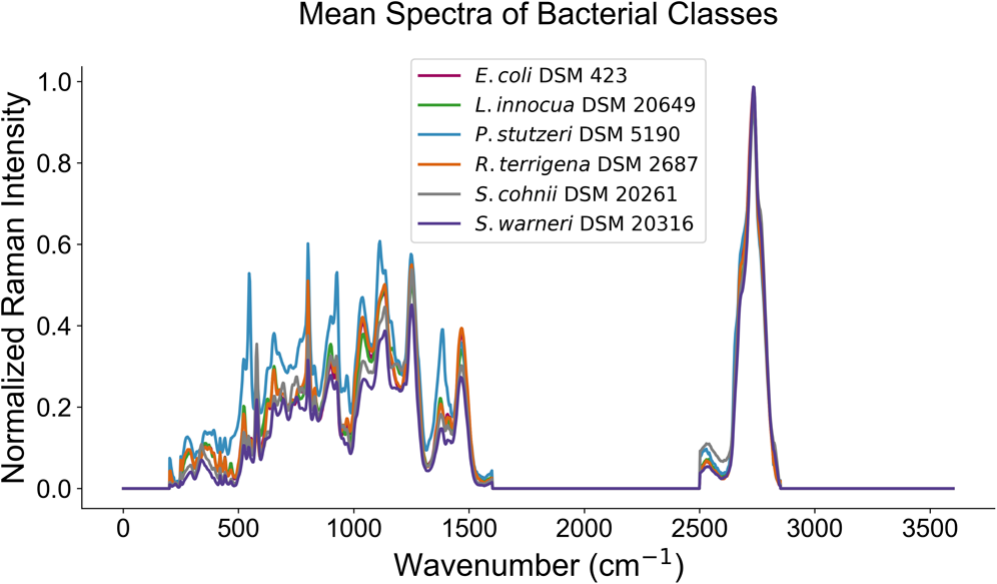
Normalized mean spectra of bacterial classes. The spectra for six bacterial classes are displayed. While some distinct features are visible, significant spectral overlap is observed, indicating similarities among the classes across the wavenumber range.

To ensure compatibility with the pretrained model’s architecture, the bacterial spectra underwent additional preprocessing steps. These steps included linear interpolation, zero padding, and the adjustment of negative intensity values to zero. These adjustments were applied conservatively to align the data shape and format with the original training set while preserving essential spectral features and minimizing changes to the original information content. The detailed steps for the preprocessing of these spectra can be found in the Supporting Information (C).

### Deep Learning Model

A one-dimensional convolutional neural network (1D-CNN) was used to pretrain the deep learning model on artificial spectra; this pretrained model is hereafter referred to as SimNet. The model architecture consists of four convolutional layers with an increasing number of feature-extracting filters, followed by a dense layer and a final classification layer as shown in Figure 7 (SimNet). A similar model is trained from scratch for six-class classification on the experimental bacterial data set, serving as a reference for comparison to the transfer-learning models used in subsequent steps.

**Figure 7:**
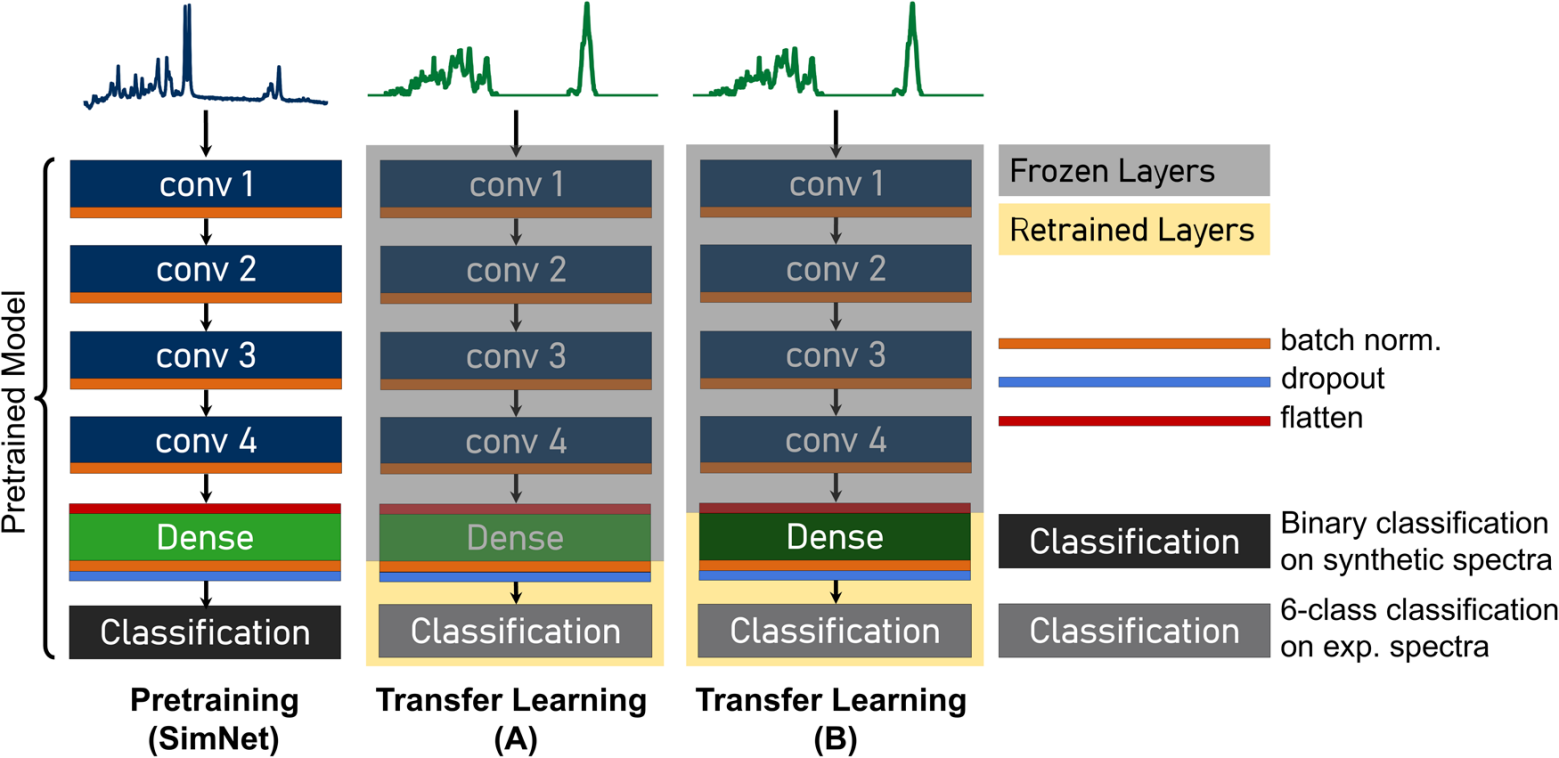
Overview of the transfer-learning methodology. (SimNet) A four-layer deep convolutional neural network (CNN) is pretrained on synthetic spectra. (A) During fine-tuning, the pretrained model’s convolutional layers are frozen, and only the new classification layer is trained on experimental bacterial spectra. (B) For further optimization, a deeper and much smaller dense layer is unfrozen and retrained along with the classification layer. Batch normalization and flatten operations are retained throughout all phases.

The hyperparameters of the model were tuned using Keras-Hyperband Tuner^46,47^ to optimize performance through systematic exploration. These included number and size of filters in each convolutional layer, learning rate, dropout rate, optimizer and activation function. When the pretrained model was subsequently modified for transfer learning, it was again subjected to a similar hyperparameter tuning process, excluding filters of the convolutional layers, to ensure consistent optimization and achieve maximum performance.^48^ The parameter space for the hyperparameter tuning can be found in the Supporting Information (D).

### Model Configurations

Summary of architecture and training configurations for the binary classification pretraining model and the multiclass bacterial classification model trained from scratch is given in Table 1. Both models are based on 1D convolutional neural networks (CNNs) with variations in activation functions, output layers, loss functions, and training parameters to suit their respective tasks and tuning results.

**Table 1:** Summary of Model Architecture and Parameters after Tuning

	binary pretraining model (SimNet)	multiclass bacterial classification model (from scratch)
input shape	(3601, 1)	(3601, 1)
conv Block 1	32 filters, kernel size 15, stride 3, activation = ReLU	32 filters, kernel size 7, stride 3, activation = SELU
conv Block 2	64 filters, kernel size 15, stride 3, activation = ReLU	64 filters, kernel size 7, stride 3, activation = SELU
conv Block 3	128 filters, kernel size 15, stride 3, activation = ReLU	128 filters, kernel size 3, stride 3, activation = SELU
conv Block 4	256 filters, kernel size 15, stride 3, activation = ReLU	256 filters, kernel size 3, stride 3, activation = SELU
pooling	none	none
flatten	yes	yes
dense layer	32 units, ReLU, dropout = 0.4 [10 units in TL config]	320 units, SELU, BatchNorm, dropout = 0.2
output layer	2 units, sigmoid	6 units, softmax
loss function	binary crossentropy	categorical crossentropy
optimizer	RMSprop (lr = 0.0001)	adagrad (lr = 0.001)
batch Size	32	128
epochs	50	as configured (e.g., 100–200 with early stopping at patience = 30)
callbacks	earlystopping (patience = 8), ReduceLROnPlateau	earlystopping (patience = 30), ReduceLROnPlateau
metric	binary accuracy	categorical accuracy
cross-validation	5-fold	batchout CV

### Performance Metrics and Cross-Validation Scheme

For the synthetic data set, which involved binary classification, a 5-fold cross-validation approach was used. The model’s performance was evaluated using the average balanced accuracy (BAC) across the 5 folds. Although the class distribution in this task was relatively balanced, balanced accuracy was chosen for consistency across different training tasks and to account for any minor differences between classes. Balanced accuracy is calculated as the average of sensitivity and specificity, where sensitivity measures the proportion of actual positives correctly identified, and specificity measures the proportion of actual negatives correctly identified.

For the experimental data set involving classification of six bacterial species, a batch-out cross-validation scheme, using nine bacteria batches within the data set, was employed for the model trained from scratch and the transfer-learning model after modification. This approach was adopted to ensure a thorough evaluation of the model’s generalization capabilities by accounting for biological variability across batches of the same species. In each fold, six batches were used for training, two for validation, and one for testing—exposing the model to diverse biological variations throughout the evaluation process. The average balanced accuracy of test samples across all folds was used as the final performance metric.

### Transfer-Learning Methodology

A transfer-learning approach was devised to leverage the feature representations learned by a model pretrained on synthetic spectral data. This model—a 1D convolutional neural network (SimNet) was trained on binary classification of artificial spectra involving 11,847 unique molecule species (6057 labeled as “alcohol” and 5790 as “aromatic secondary amine”). In the transfer-learning phase, SimNet was adapted for multiclass classification on the experimental bacterial Raman data set comprising six distinct species, as outlined in the preceding sections.

The transfer-learning procedure involved two stages. First, the entire pretrained model was imported, and all layers were frozen except for a newly added output classification layer tailored to the six bacterial classes. Only this new layer was trained initially, allowing the model to adapt to the new task while retaining the learned spectral features from the synthetic domain. This setup is illustrated in Figure 7 (Transfer Learning (A)).

Once the new classification layer achieved stable performance, a gradual unfreezing procedure was applied: the layers closest to the classification head were unfrozen first, and the model was fine-tuned end-to-end with a reduced learning rate. This allowed the model to adjust deeper feature representations to the characteristics of the experimental data set while avoiding catastrophic forgetting. This second stage is illustrated in Figure 7 (Transfer Learning (B)). The goal of this stepwise approach was to maximize knowledge transfer from the artificial domain while optimizing performance for real biological data.

This progressive unfreezing allowed for a controlled adaptation of the pretrained model to the new data set while preserving the essential feature extraction capabilities learned during pretraining. To assess the impact of leveraging synthetic data in this setup, the results were compared with those obtained from model trained from scratch (i.e., without transfer learning). Additionally, these results were compared with traditional chemometric techniques, specifically a PCA-LDA pipeline, where Principal Component Analysis (PCA) was used to reduce the dimensionality of the spectra while retaining 95% of the variance, and Linear Discriminant Analysis (LDA) was then applied for supervised feature extraction. The number of PCA components varied depending on the data set structure, while LDA reduced the data to a maximum of five components (*n* – 1, for six-class classification). The resulting features were used to train a logistic regression classifier, and performance was evaluated using same batch-out-cross-validation scheme.

Throughout the transfer learning process, the CNN’s feature extraction part, specifically the convolutional layers, remained frozen to preserve the core features learned from the synthetic data. This approach provided insights into how much fine-tuning was required for the classification layers to adapt to the new data and how it performed compared to models built without pretraining or with conventional feature extraction techniques like PCA and LDA.

## Results and Discussion

The results of all models, along with their training parameters, are shown in Table 2. The corresponding confusion matrices are given in Figure 8 for comparison. All results represent the mean balanced accuracy of the test batches across 9 folds of the experimental bacterial data set, in the batch-out-cross-validation scheme.

**Figure 8:**
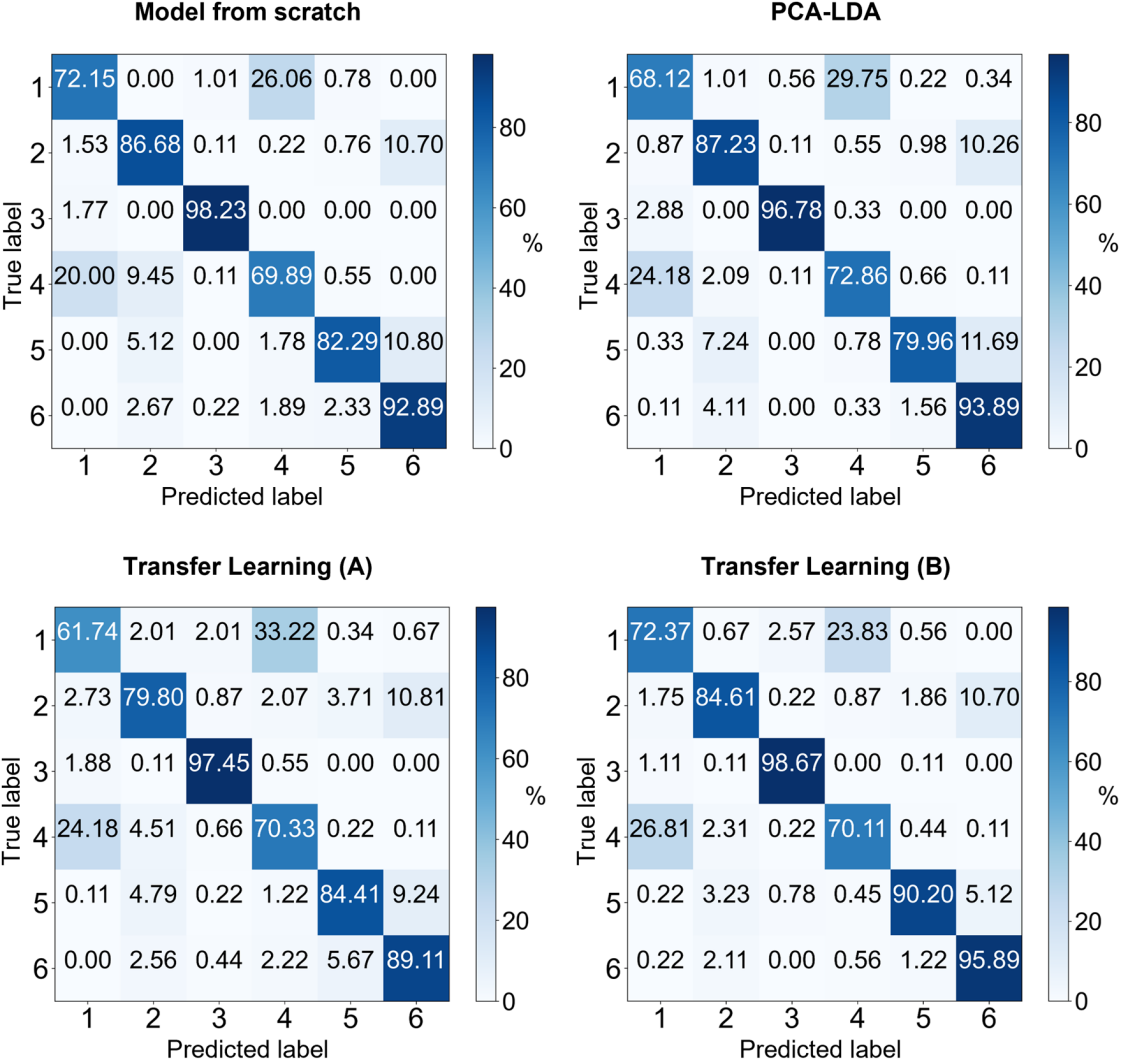
Confusion matrices for the four evaluated models: Model trained from scratch, transfer learning A, transfer learning B, and PCA-LDA baseline. Each matrix shows the classification performance across the six bacterial classes in the test.

**Table 2:** Summary of Model Performance Metrics

			transfer learning	
	model from scratch	PCA-LDA	(A)	(B)
balanced accuracy	83.66% ± 6.90%	83.09% ± 8.14%	80.47% ± 6.51%	85.30% ± 5.67%
training parameters	3.74 M	240 ± 14^a^	2246	98k

a Number of Principal Components.

Before using the transfer learning methods, the performance of SimNet was evaluated on synthetic spectra to assess its ability to perform the intended task. The model was trained to perform binary classification between two functional groups: Alcohol and Aromatic Secondary Amine (Figure 5). However, since contributions from all other functional groups are present in the spectra as well, the models must learn not only to extract the features relevant to the two classes but also learn to disregard the irrelevant features associated with these other functional groups. Using a 5-fold cross-validation scheme, the model achieves 90.14% ± 0.34% balanced accuracy on the synthetic data set. In comparison, a PCA-LDA for this simulated data set reaches 82.43% ± 1.05% under the same evaluation setup. Although the final transfer-learning models do not outperform PCA-LDA on the bacterial classification task, the CNN demonstrates substantially stronger performance during pretraining. This indicates that the network learns expressive, discriminative features from the simulated spectra—features that may not be linearly separable or accessible through variance-based methods like PCA. These results confirm the model’s ability to distinguish between classes in a controlled setting, while also highlighting its capacity to reject overlapping functional group labels, manage noise and background interference, and remain robust to peak broadening effects introduced during simulation. Together, these characteristics support the CNN’s role as a suitable backbone for transfer learning, even when downstream improvements are modest.

However, it is important to note that these results reflect performance in the pretraining domain only and are not directly comparable to the results presented later in this section, which focus on the real experimental data set. The primary purpose of this evaluation was to confirm that the pretrained model provides a strong and generalizable feature extractor to be leveraged through transfer learning.

Next, a CNN model was trained from scratch on the bacterial Raman spectra data set. This approach resulted in an accuracy of 83.66% ± 6.90% but required over 3.5 million parameters, indicating that while the model could learn the task, it was computationally intensive.

Subsequently, SimNet (the pretrained model from the synthetic spectra) was fine-tuned on the bacterial data. By training only the newly added classification layer, as shown in Figure 7 (Transfer Learning (A)), with approximately 2246 parameters, the model achieved an accuracy of 80.47% ± 6.51%, demonstrating that the features learned during pretraining were transferable and useful for the new classification task. Importantly, this also validates the efficacy of using synthetic spectra as a viable pretraining data set, illustrating that the model can successfully leverage the knowledge gained from synthetic data for real-world applications.

When the last dense layer was also retrained, as shown in Figure 7 (Transfer Learning (B)), the model’s accuracy increased to 85.30% ± 5.67% with only 98,336 parameters, a significant reduction compared to the model trained from scratch and with around 2% improvement in the balanced accuracy.

A summary of performance for all models is given in Figure 9A and the comparison of training parameters across models is shown in Figure 9B.

**Figure 9:**
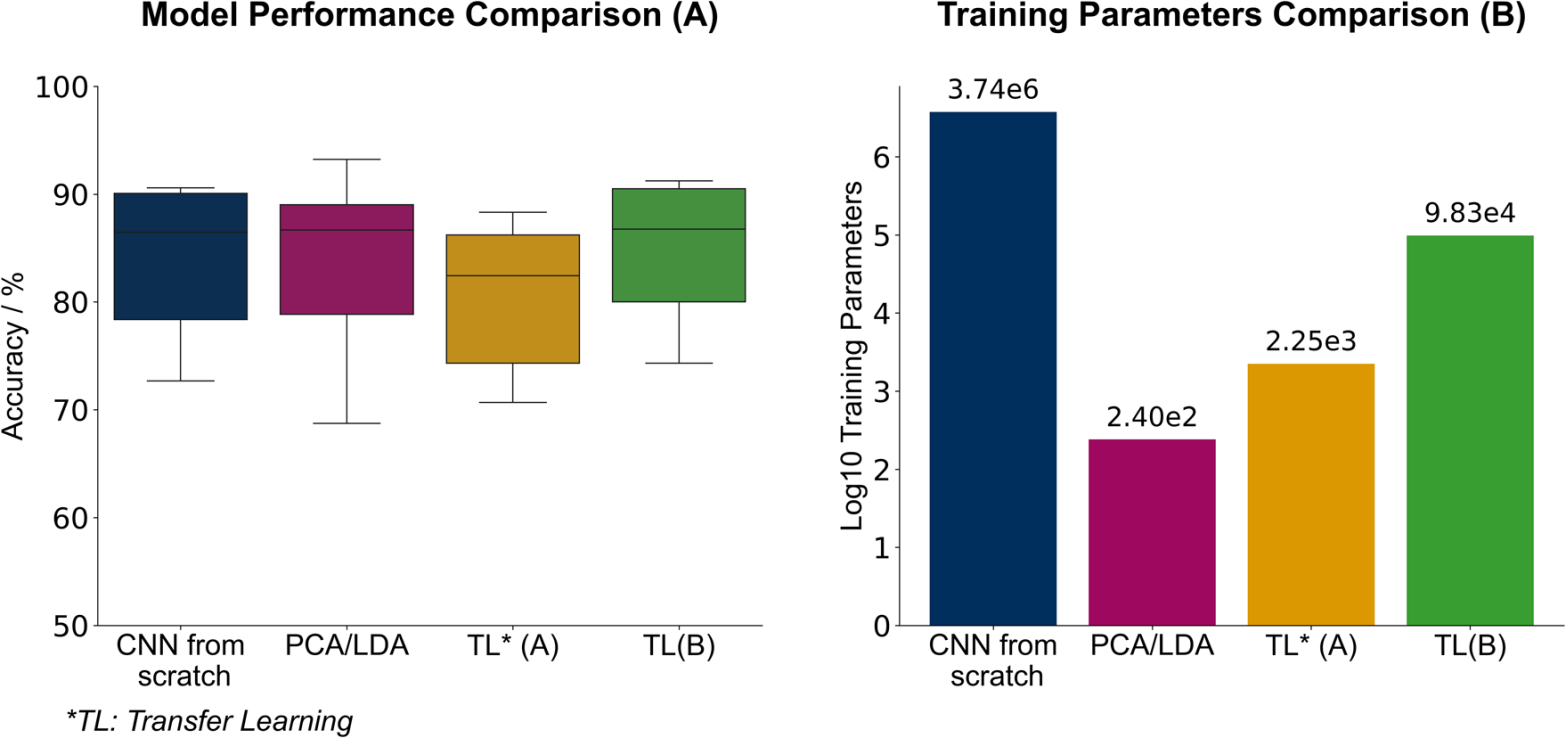
(A) Boxplot illustrating the mean balanced accuracy percentages (*x̅* ± σ) of different models: CNN from scratch: 83.66 ± 6.90%, PCA-LDA: 83.09 ± 8.14%, transfer learning (A): 80.47 ± 6.51%, and transfer learning (B): 85.30 ± 5.67. Although the final transfer learning results show the highest mean accuracy, statistical analysis did not reveal significant differences between models. (B) Bar plot comparing the log_10_-scaled training parameters, highlighting significant efficiency differences: TL­(A) with 2246 parameters and TL­(B) with 98k parameters achieve competitive accuracy despite CNN from scratch requiring 3.74 M parameters.

A statistical analysis of the balanced accuracy distributions using the Kolmogorov–Smirnov (KS)^49^ and Mann–Whitney *U* ^50^ tests revealed no significant differences between the models (*p* > 0.05 for all pairwise comparisons). The Mann–Whitney *U*-test is a nonparametric test suitable for comparing the medians of two independent groups without assuming normality in the data. It can highlight whether one model consistently outperforms another based on central tendencies. In contrast, the KS test is used to compare the entire distributions, in shape and spread, of model performance. Together, these tests can provide sound statistical assessment of models trained with and without transfer learning. Our tests indicates that while performance variations exist, they are not statistically significant. However, Transfer Learning (A) and Transfer Learning (B) achieve these results with significantly lower computational costs—2246 and 98k parameters, respectively—compared to 3.74 million parameters required by the model trained from scratch. This highlights the efficiency of transfer-learning approaches without compromising performance.

Finally, a comparison with the traditional PCA-LDA method was conducted. An analysis of the confusion matrices (as shown in Figure 8) across models trained from scratch, those utilizing transfer learning, and the PCA-LDA approach revealed a consistent pattern of misclassified samples, with the majority arising from DSM 423 and DSM 2687 shown by the class labels 1 and 4 in Figure 8. Both are Gram-negative rods within the order *Enterobacterales*: belongs to the genus Escherichia, while is part of the genus *Raoultella* (previously classified under the genus *Terrigena* until 2001).^51^ These genera share substantial phenotypic similarities, and some strains have been reported to appear nearly identical, leading to misclassification in routine bacterial identification tests.

Both bacteria are oxidase-negative, facultative anaerobes that ferment lactose with gas production, making them difficult to distinguish in standard coliform assays and traditional culture-based techniques.^52^ For instance, both can yield positive results in the fecal coliform test (gas from lactose at 44.5 °C), despite being of fecal origin and being environmental. This overlap results in colonies that are nearly indistinguishable on differential media and in basic biochemical assays.^53^

Such biological similarity explains the persistent confusion observed across all classification methods. The intergroup variations between and are insufficiently large relative to the intragroup variations,^54^ making these classes particularly challenging to distinguish. Notably, this consistent misclassification pattern underscores the robustness of the features learned during the pretraining phase: despite reduced training effort and fewer parameters, transfer learning models achieved performance comparable to models trained from scratch and the classical PCA-LDA approach.

The performance of PCA-LDA and models trained from scratch was found to be comparable to that of transfer-learning models pretrained on the synthetic data set when evaluated on this particular data set of bacterial spectra. Although relatively large by experimental standards, the data set was intentionally selected for the proof-of-concept phase to establish the maximum achievable performance under optimal conditions. This baseline was necessary to set expectations for future comparisons when applying transfer-learning methodologies to smaller, more limited data sets.

While models trained from scratch have shown similar performance to PCA-LDA and transfer-learning models, their computational cost is significantly higher. Training models from scratch requires substantial time and computational resources, particularly as the model complexity increases with more parameters. In contrast, transfer-learning models, which leverage pretrained models, offer a much more computationally efficient approach without compromising performance.

The intended application of this transfer-learning methodology will likely involve much smaller data sets. Future experiments will explore how performance scales with reduced data sizes. However, before evaluating on smaller subsets, cautious sample size planning is necessary to define what constitutes a meaningful “small” data set. Especially within biological contexts, uncontrolled down-sampling can lead to unrepresentative or unstable subsets, regardless of class balance. While stratified splitting can maintain class proportions, and balanced accuracy accounts for uneven distributions, a poorly sized subset may still lead to unreliable performance estimates. Careful sample planning will therefore be needed to ensure that reduced data subsets remain statistically valid and representative.

The success of this approach further highlights the flexibility and control afforded by generating synthetic data using QC methods. Since the entire pretraining process is based on systematically generated data, the workflow can be easily adapted to suit different tasks and experimental conditions. The number of synthetic spectra generated can be modified to adjust the data set size or diversity, and the selection of initial molecules used for spectral calculations can be tailored to better match the target domain or to broaden the model’s generalization capabilities. Additionally, synthetic spectra can be customized by introducing various types of artifacts, such as noise or background signals, to better simulate real-world experimental conditions, thereby enhancing the robustness of the pretraining process.

This flexibility not only allows for tailoring synthetic data to specific tasks but also enables iterative refinement of the model. By varying initial molecules sets or adjusting the conditions for spectra generation, the pretraining phase can be systematically optimized for various applications, increasing the effectiveness of the transfer-learning process. Overall, the ability to generate and fine-tune synthetic data provides extensive opportunities to explore how different configurations impact model performance, enabling a versatile and customizable framework for a wide range of classification tasks.

Unlike traditional chemometric approaches such as PCA-LDA, which cannot leverage external data sets for representation learning, deep learning models benefit directly from large-scale synthetic pretraining. Methods like PCA and LDA are inherently data set-specific and do not retain transferable features; their statistical assumptions limit them to the structure of the data set they are trained on. In contrast, the CNN-based transfer-learning pipeline developed here enables representation learning from simulated data and generalization to new, unseen experimental domains. This capacity to scale with data and adapt across tasks makes transfer learning uniquely suited for low-data scenarios where traditional methods offer no analogous pretraining mechanism.

In summary, while PCA-LDA, transfer-learning models, and models trained from scratch have shown comparable performance on large data sets, training from scratch involve significantly higher computational costs. As future work transitions to testing smaller data sets, transfer-learning models are expected to offer a more practical and efficient solution. The synthetic spectra-based pretraining methodology not only demonstrates its utility but also provides the flexibility to be adapted and optimized for different tasks, which will be key in exploring its full potential.

## Conclusions

This study demonstrated the effectiveness of transfer-learning with synthetic Raman spectra generated via semiempirical quantum chemistry methods for bacterial classification. Transfer-learning models achieved comparable performance to those trained from scratch, with balanced accuracies exceeding 84%, while significantly reducing trainable parameters and computational costs. Leveraging synthetic data for pretraining addresses the challenge of limited experimental data sets, particularly in resource-constrained environments. The flexibility of synthetic data generation allows for the customization of data sets to suit specific tasks, making the approach more robust. This scalable and flexible framework offers a promising solution for a wide range of spectral analysis tasks.

Beyond spectral analysis, this approach underscores the broader potential of synthetic data-driven transfer learning in scientific fields where large experimental data sets are scarce. By bridging the gap between theoretical modeling and real-world applications, it enables more scalable and accessible AI-driven solutions. Future efforts will focus on applying this approach to smaller data sets where current methods perform far worse as well as to more diverse data sets to evaluate its adaptability and broader applicability.

## Supplementary Material



## Data Availability

The pretraining data set, along with the associated scripts and simulated spectra used in this study, are available on Zenodo at: 10.5281/zenodo.15482140 The repository includes: The pretraining data set used for model development and evaluation. The simulated line spectra generated using semiempirical quantum chemistry methods. Python scripts used for spectrum simulation, background augmentation, artifact addition, and model training. Pretrained model (SimNet). Other relevant information, such as line spectra calculation model, hyperparameters, data splits, and evaluation procedures, is described in the corresponding sections of the manuscript, the Supporting Information sections and in the citations.

## Notes

Mathematical derivation for calculating the Voigt profile used in spectra broadening pipeline, the detailed list and distribution of all functional groups present in the synthetic spectra dataset (Figure S1), the preprocessing steps applied to the experimental bacterial spectra to ensure compatibility with the pretrained deep learning model, and the hyperparameter tuning parameters used for optimizing the 1D convolutional neural network (PDF)
