# Supplementary material for: Transfer-Learning Deep Raman Models Using Semiempirical Quantum Chemistry
Source: J Chem Inf Model. 2025 Jun 18;65(13):6632–43. doi: 10.1021/acs.jcim.5c00513 (PMC12264939; doi:10.1021/acs.jcim.5c00513)
Supplement: Supplementary file 1 [file ci5c00513_si_001.pdf]

Supporting Information

Transfer Learning Deep Raman Models using Semi-Empirical Quantum Chemistry

Jawad Kamran[1,2], Julian Hniopek[1,2], Thomas Bocklitz[1,2,*]

[1] Institute of Physical Chemistry, Friedrich Schiller University Jena, Helmholtzweg 4, 07743 Jena, Germany

[2] Department of Photonic Data Science, Leibniz Institute of Photonic Technology, Albert-Einstein-Straße 9, 07745 Jena, Germany

Corresponding author: Thomas Bocklitz; E-mail: thomas.bocklitz@uni-jena.de

Contributing authors: JK: jawad.kamran@leibniz-ipht.de; JH: julian.hniopek@uni-jena.de

Supporting Information contains: 4 pages and 1 figure.

A. Mathematical derivation for calculations of Voigt profile

The Voigt profile, denoted as $V(x; \sigma, \gamma)$, is defined as the convolution of a Gaussian function $G(x; \sigma)$ and a Lorentzian function $L(x; \gamma)$. Mathematically, it can be expressed as follows:

$$V\,(x;\,\sigma,\gamma) = \int_{-\infty}^{\infty} G(x - t;\,\sigma) \cdot L(t;\,\gamma)\,dt \tag{1}$$

$$= \int_{-\infty}^{\infty} \frac{\sigma}{(x-t)^2 + \sigma^2}\,dt \tag{1.1}$$

In Eq.1.1, x is the independent variable, σ represents the standard deviation of the Gaussian component, and γ is the half-width at half-maximum (FWHM) of the Lorentzian component.

To simulate the broadening effects, the line spectra obtained from the computational methods were broadened using the Kielkopf approximation [39] of the Voigt profile function as given in Eq. 2. For a set value of full width at half maximum (FWHM) (denoted as f), the parameters of Voigt profile were calculated as:

$$f_V = 0.5346 \cdot f_L + \sqrt{0.2166 \cdot {f_L}^2 + {f_G}^2} \tag{2}$$

whereas, for Gaussian and Lorentzian profile the FWHM is given as:

$$f_G = 2 \cdot \sigma \cdot \sqrt{2\ln(2)}$$

$$f_L = 2 \cdot \gamma$$

Setting $k = \frac{f_L}{f_G}$ in Eq.2:

$$f_G = \frac{f_V}{\left(0.5346 \cdot k + \sqrt{0.2166 \cdot k^2 + 1}\right)}$$

$$f_L = k \cdot f_G$$

Whereas for Gaussian: $\sigma = f_G \cdot 2.3458$ and for Lorentzian: $\gamma = k \cdot \frac{f_G}{2}$.

B. Complete list of functional groups present in the synthetic dataset and their distribution

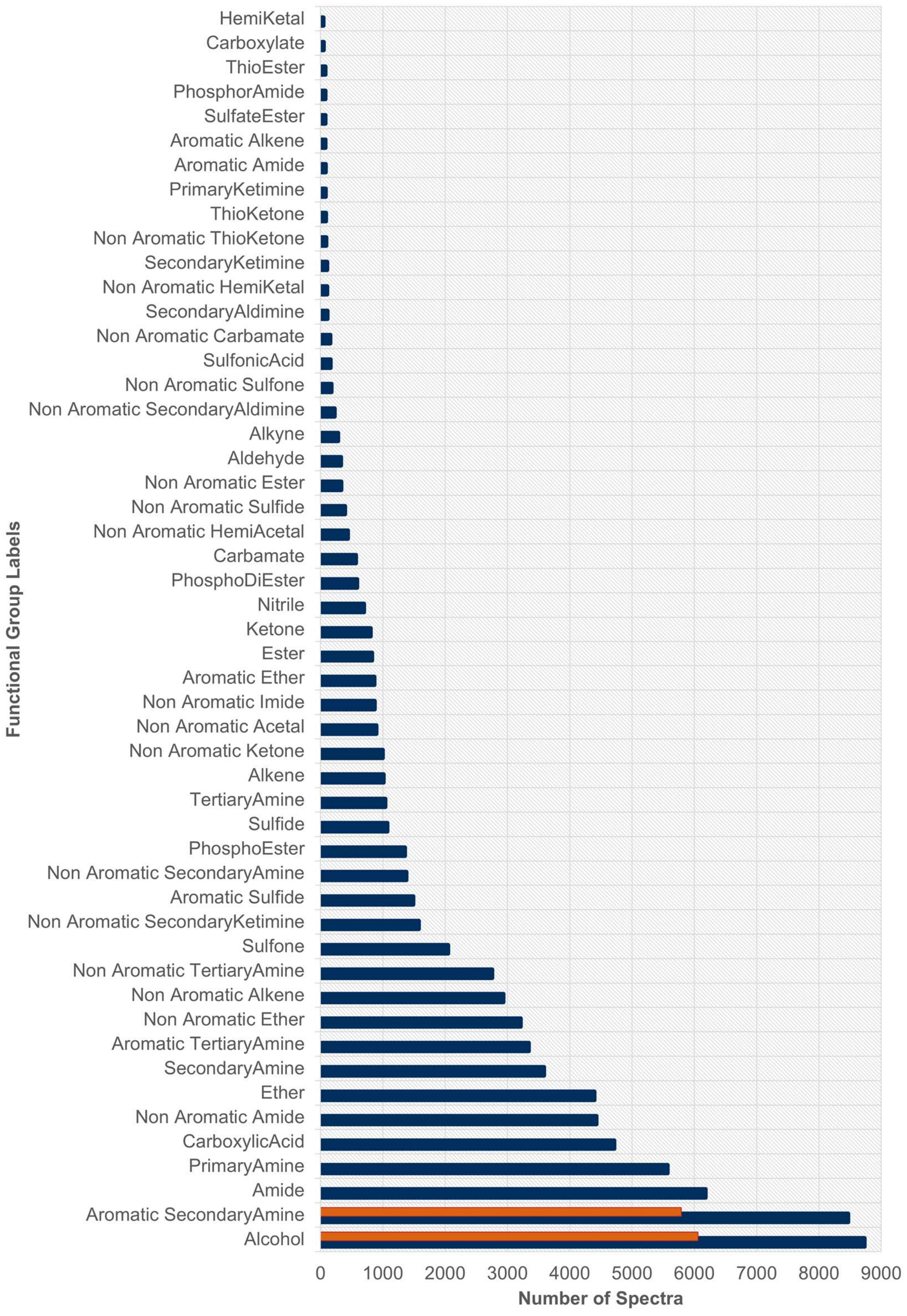

Figure S1: Distribution of functional groups in the synthetic spectral dataset. Each bar indicates the number of spectra which contains the given functional group label. The two target classes used for pretraining—Alcohol and Aromatic Secondary Amine—are highlighted in orange, representing the number of mutually exclusive spectra assigned to each class in the dataset. The overall distribution shows substantial class imbalance, with many functional groups being underrepresented.

C. Preprocessing of Bacteria spectra

Spectral data were interpolated from an original wavenumber range of 401–3050 cm^{-1} with 3 cm^{-1} steps to a finer resolution of 1 cm^{-1} using linear interpolation. Missing wavenumbers between 1802–2702 cm^{-1} (Silent region) were assigned zero values. Negative values in the spectra were set to zero. To standardize input dimensions, zero-padding (201 and 750 points) was applied at both ends.

D. Hyperparameter tuning parameters

The 1D Convolutional Neural Network (CNN) was optimized using Keras-Hyperband tuning to improve classification performance. The search space included:

Kernel size (3–20)

Stride (1–5)

Activation functions (ReLU, ELU, SELU, Swish)

Optimizers (Adam, RMSprop, SGD, Nadam, Adagrad)

Dropout rate (0–0.8)

Dense layer units (32–512)

Learning rate (10^{-1} to 10^{-5})

The best hyperparameters were selected based on validation accuracy after training on 5,000 spectra with a 70:30 split. The optimized model was saved for further evaluation.
